# Continuous Activation of C/EBPβ Transcription Factor Prevents Fibrosis Resolution After Alcohol Cessation

**DOI:** 10.1016/j.jcmgh.2025.101525

**Published:** 2025-04-26

**Authors:** Michael Schonfeld, Kruti Nataraj, Samson Mah, Steven Weinman, Irina Tikhanovich

**Affiliations:** 1 Department of Internal Medicine, University of Kansas Medical Center, Kansas City, Kansas; 2 Kansas City VA Medical Center, Kansas City, Missouri

**Keywords:** Abstinence, Epigenetic Changes, Collagen Degradation, Endothelial Dysfunction

## Abstract

**Background & Aims:**

Abstinence is an important therapeutic intervention for patients with alcohol-associated liver disease (ALD). However, fibrosis improvement after cessation is not uniform and some patients do not improve.

**Methods:**

Mice were fed high-fat diet with 20% alcohol in the drinking water for 20 weeks (ALD) followed by 4 weeks of chow diet with plain water (resolution). scATAC-seq dataset was analyzed using Signac R package. *Cebpb* floxed mice received AAV-TBG-Cre or AAV-control at the time of alcohol cessation. Hepatocyte-macrophage and endothelial cell-hepatocytes crosstalk was investigated using a Transwell coculture system. To test the role of angiopoietin mice were treated with recombinant angiopoietin-1, 1 week after alcohol cessation.

**Results:**

We analyzed differentially accessible regions in hepatocytes from control, ALD, or 4 weeks post alcohol cessation mice and identified transcription factors activated in ALD that remained activated after alcohol withdrawal. The top hit was CCAAT enhancer binding protein beta (C/EBPβ). We found that hepatocyte-specific *Cebpb* knockout at the time of alcohol cessation promoted fibrosis resolution. The resolution was mediated by altered hepatocyte-macrophage crosstalk. C/EBPβ suppressed the expression of CYP3A family of enzymes in hepatocytes and downstream macrophage collagen degradation ability. *Cebpb* knockout in hepatocytes promoted a proresolving phenotype in liver macrophages. We further identified upstream events leading to persistent C/EBPβ activation. C/EBPβ was induced by alcohol-mediated endothelial changes during ALD development and resolution. Restoring endothelial cell function with angiopoietin-1 supplementation reduced C/EBPβ and promoted fibrosis resolution.

**Conclusions:**

Taken together, alcohol-induced C/EBPβ activation is a key driver of poor disease resolution in ALD and a promising target for patients who fail to recover after alcohol abstinence.

## Graphical abstract

**Figure:**
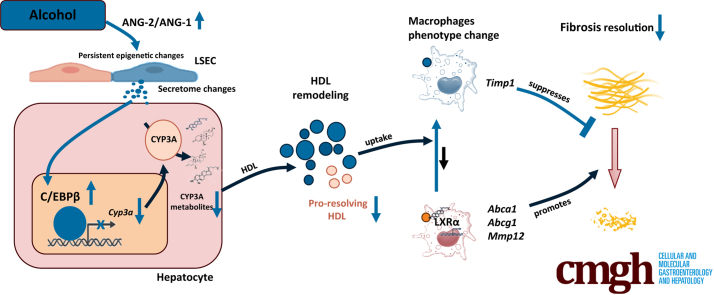

**Summary**

Fibrosis resolution after alcohol cessation is prevented by endothelial cell–mediated up-regulation of hepatocyte C/EBPβ. C/EBPβ suppresses CYP3A in hepatocytes thus altering HDL proresolving properties. Loss of C/EBPβ stimulates macrophage proresolving phenotype and collagen remodeling promoting fibrosis resolution.

This article has an accompanying editorial.

Alcohol-associated liver disease (ALD) encompasses a spectrum of disorders that commonly progress from steatosis to steatohepatitis with fibrosis and ultimately to cirrhosis. Cirrhosis is the ninth leading cause of death in the United States and about 35%–50% of cirrhosis deaths are alcohol related.^1^^,^^2^ Based on studies in multiple liver diseases, removing the harmful agent typically stops the progression and promotes resolution, even in advanced disease.^3^^,^^4^ Alcohol abstinence is thus the most critical therapeutic intervention for patients with ALD. However, fibrosis improvement after drinking cessation is not uniform and some patients progress to cirrhosis or develop decompensation even while abstinent.^1^^,^^5^^,^^6^ Understanding the molecular mechanisms of ALD resolution could lead to better and more frequent resolution in patients with ALD who stop drinking.

Fibrosis resolution after alcohol cessation is not well studied, mostly caused by the absence, until recently, of animal models that can induce clinically relevant fibrosis. However, data from nonalcohol disease models suggest that fibrosis resolution involves apoptosis of activated hepatic stellate cells (HSCs) or HSC reversal to a quiescent state,^7^ collagen removal by increased matrix metalloproteinase (MMP) dependent degradation,^8^, ^9^, ^10^, ^11^ and uptake of degraded collagen by macrophages.^12^ Our data in mouse models indicate that in ALD, fibrosis resolution on alcohol cessation is extremely slow and this is different from the situation in other models of liver fibrosis induced by chronic liver injury.^13^ One possible explanation is that alcohol induces changes in the liver that persist after alcohol cessation (eg, epigenetic changes in hepatocytes), as evidenced by our assay for transposase-accessible chromatin with sequencing (ATAC-seq) data, which prevents collagen degradation normally occurring during disease resolution. Recently we identified the H3K4-specific demethylases KDM5B and KDM5C as mediators of alcohol-induced epigenetic changes that prevent collagen degradation and fibrosis resolution after alcohol exposure.^13^ However, KDM5 demethylase chromatin binding requires recruitment by specific transcription factors (TF), because demethylases themselves do not have DNA binding specificity. In this work we identified the CCAAT enhancer binding protein beta (C/EBPβ) TF as a potential mediator of alcohol-induced epigenetic changes.

C/EBPβ is a liver enriched TF. Its protein level is low/undetectable under control conditions, but it gets rapidly induced after liver injury as part of a regeneration program.^14^, ^15^, ^16^, ^17^, ^18^, ^19^ C/EBPβ is activated by a variety of signals that play a role in regulation of multiple inflammatory pathways.^14^, ^15^, ^16^^,^^20^, ^21^, ^22^ It can be activated by interleukin (IL)17A, IL10, IL27, HGF, and IGFBP-1, as well as other factors including caloric restriction, β-adrenergic receptor signaling, and cAMP.^16^^,^^22^, ^23^, ^24^, ^25^, ^26^ Previous studies have reported that C/EBPβ dysregulation is involved in cancer,^20^^,^^27^ lifespan shortening,^14^^,^^28^ and acute-on-chronic liver failure.^29^ Recently, we identified that in the liver, C/EBPβ activation is mediated by endothelial dysfunction and consequent dysregulation of angiopoietin (ANG-1/ANG-2) signaling.^29^

Here we report that ALD-associated endothelial cell changes induce C/EBPβ up-regulation in hepatocytes, and this promotes metabolic dysregulation and liver fibrosis. Persistent continuous C/EBPβ upregulation after alcohol cessation subsequently prevents fibrosis resolution in part through altered hepatocyte-macrophage crosstalk. Restoring endothelial cell function by ANG-1 supplementation or knocking out *Cebpb* promotes fibrosis resolution, suggesting that the ANG-C/EBPβ axis is a promising target for enhancing ALD resolution.

## Results

### C/EBPβ Was Persistently Activated by Alcohol

**Figure 1:**
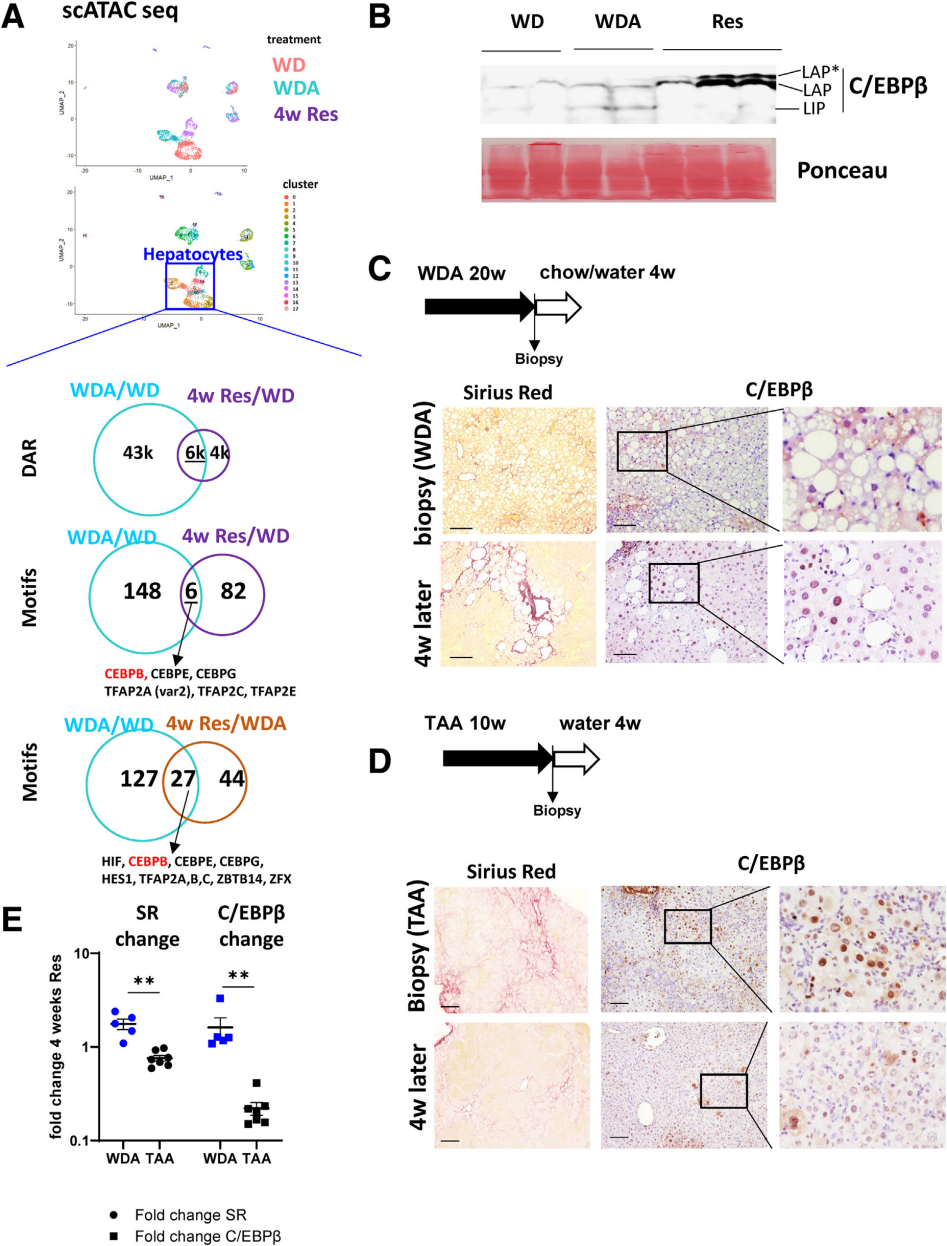
**C/EBPβ elevation correlates with poor fibrosis resolution in ALD.** (*A*) scATAC-seq analysis (BioProject PRJNA1022784). Single cell ATAC-seq analysis of liver cells from mice fed WD for 16 weeks (n = 1), WDA for 16 weeks (n = 2), and 16 weeks of WDA followed by 4 weeks of resolution (Res, n = 1). UMAP clustering of liver cells and Motif enrichment analysis. Hepatocyte clusters unique for WD, WDA, and Resolution conditions were used for motif enrichment analysis. (*B*) C/EBPβ levels were assessed by Western blot analysis in mice fed WD, WDA, or at 4 weeks after alcohol cessation (Res). (*C, D*) Mice were fed WDA or TAA in the drinking water to induce liver fibrosis. Sirius red staining and C/EBPβ staining in these mice. (*E*) Fold change in C/EBPβ staining and Sirius red staining at 4 weeks of resolution compared with biopsy for each mouse. Scale bar 100 μm. ∗∗*P* < .01.

To analyze epigenetic changes induced by alcohol exposure that persist after alcohol withdrawal we used recently performed scATAC-seq analysis.^13^ Mice were fed high-fat Western diet with alcohol in the drinking water (WDA) or plain water (WD) as a control as previously described.^30^ WDA fed mice were returned to chow diet with plain water for 4 weeks (Res) to evaluate the changes after alcohol cessation. Previously, we showed that in these mice liver steatosis was rapidly resolved but liver fibrosis did not change after stopping alcohol.^13^ Figure 1*A* shows UMAP plots with cells colored according to the treatment and assigned into clusters. We observed that there are unique hepatocyte clusters that are specific to each treatment condition. Using scATAC-seq data we performed differential accessible regions (DARs) analysis in unique hepatocyte clusters using Signac R package followed by TF motif enrichment analysis as previously described (Figure 1*A*).^31^ Among 49 thousand DARs affected by alcohol, most had reverted to control conditions after alcohol cessation. However, the change in 15% of DARs (6352 regions) persisted, suggesting that these changes could contribute to poor disease resolution. Motif analysis showed that 154 motifs were enriched in alcohol-specific DARs and 88 motifs in resolution DARs with 6 motifs common between the 2, including the C/EBPβ motif. TF C/EBPβ motif was not present in WD control DARs, whereas the motif frequency was around 5% in DARs from both alcohol and resolution condition (Padj < .05), suggesting that C/EBPβ is activated by alcohol and remains activated during resolution. Moreover, the C/EBPβ motif was enriched in Res/WDA DARs (Figure 1*A*), suggesting that C/EBPβ could be further activated by alcohol/WD withdrawal.

We next examined C/EBPβ protein expression. C/EBPβ protein levels were elevated in alcohol-fed mice (compare WDA with WD control) as previously described^32^ and further elevated after alcohol cessation in agreement with the scATAC-seq prediction (Res, Figure 1*B*). We found that the main C/EBPβ isoform that was induced during resolution was the longer isoform, liver-activating protein, but not the shorter liver-inhibitory protein isoform. Using immunohistochemistry staining we further showed increase in C/EBPβ staining in hepatocyte nuclei during ALD resolution (Figure 1*C*). We found that in agreement with previous studies^13^ 4 weeks after alcohol cessation liver fibrosis assessed by Sirius red staining was not resolved in these mice compared with the end of alcohol feeding biopsy, which correlated with an increase in C/EBPβ staining (Figure 1*C*). The strongest staining occurred in the areas of liver fibrosis. In contrast, in a model of liver fibrosis induced by thioacetamide (TAA) where C/EBPβ protein level was elevated in hepatocytes adjacent to fibrotic septa (Figure 1*D*), fibrosis levels and C/EBPβ levels were significantly down-regulated 4 weeks after TAA removal (Figure 1*D* and *E*).

Overall, we observed that C/EBPβ protein levels were elevated after alcohol cessation, and persistent C/EBPβ elevation correlated with poor fibrosis resolution in ALD model.

### C/EBPβ Prevented Fibrosis Resolution After Alcohol Cessation

**Figure 2:**
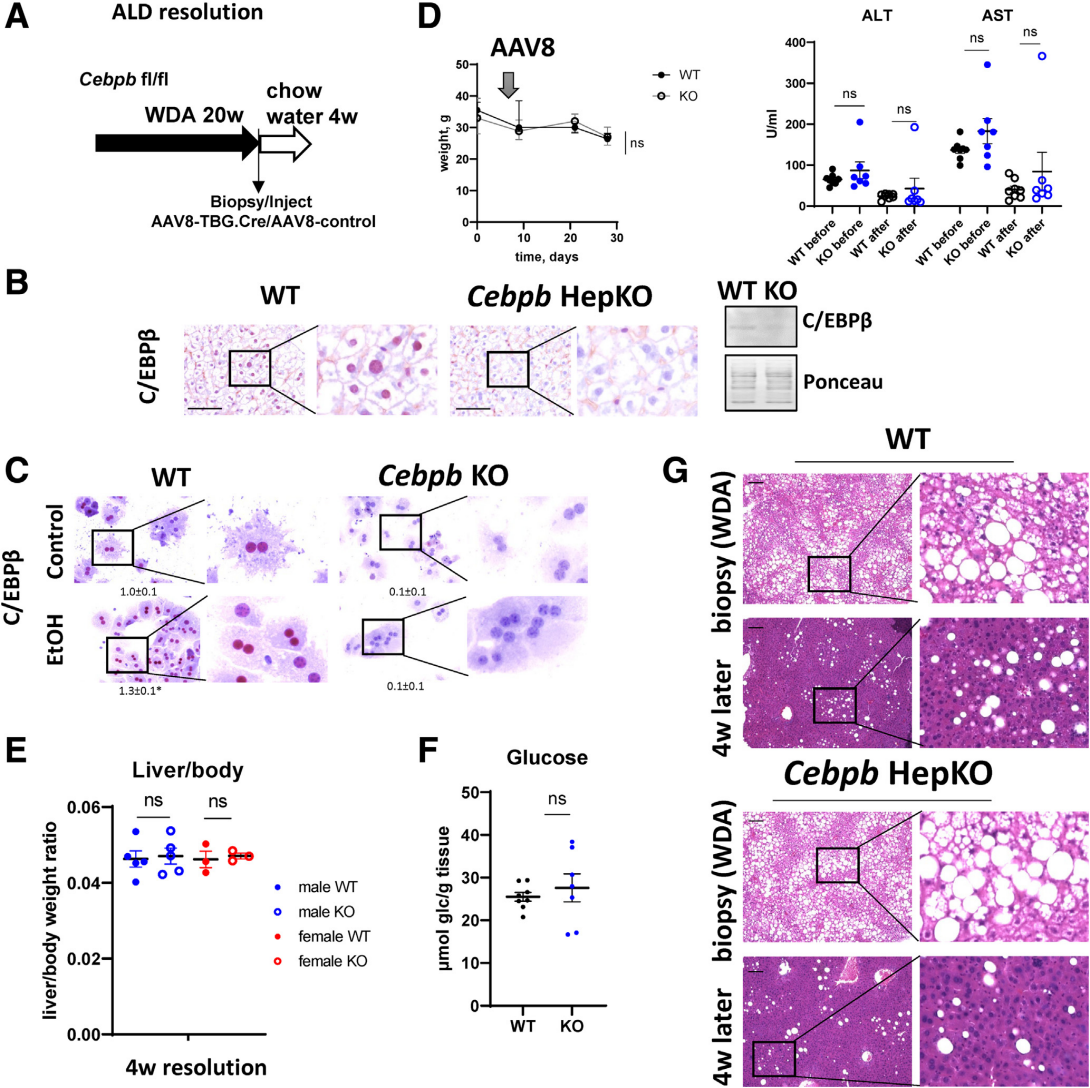
**Hepatocyte-specific *Cebpb* knockout does not affect liver injury and steatosis resolution in AD mice.** (*A*) Seven- to 8-week-old *Cebpb* fl/fl mice were fed ad libitum WDA for 20 weeks then liver biopsy was collected, and mice were placed on chow diet with plain water for total of 4 weeks. One day later after biopsy, mice received 10^11^ gc/mouse of AAV-TBG-Cre or AAV-TBG-control. (*B*) C/EBPβ staining in WT and hepatocyte-specific *Cebpb* knockout mice. (*Right*) Western blot analysis of C/EBPβ protein levels in WT and KO mice at 4 weeks after alcohol cessation. (*C*) Mouse primary hepatocytes were isolated from *Cebpb* floxed mice and treated with Cre expressing vector (*Cebpb* KO) or control (WT). Cells were treated with 50 mM ethanol for 24 hours and analyzed by immunohistochemistry. (*D*) Weight change in mice after stopping WDA. Serum alanine aminotransferase (ALT) and aspartate aminotransferase (AST) in mice at the end of WDA feeding (before) and at 4 weeks after (after). (*E*) Liver to body weight ratios at the end of the experiment. N ≥3 per group. (*F*) Liver glucose levels at 4 weeks after stopping alcohol. (*G*) Representative images of hematoxylin and eosin staining from corresponding biopsy and livers after 4 weeks of resolution. Scale bar 100 μm. ns, not significant.

We next examined the role of hepatocyte C/EBPβ in ALD liver disease resolution using a hepatocyte-specific *Cebpb* knockout (KO) mouse model (Figure 2). To do so, we fed *Cebpb* floxed mice WD with alcohol in the drinking water for 20 weeks. At the end of the feeding, we collected a small liver biopsy from each mouse to compare the fibrosis levels before and after alcohol cessation. Once the high-fat diet and alcohol were stopped, mice received adeno-associated virus (AAV)8-TBG-Cre or AAV8.TBG-control (Figure 2*A*). TBG promoter driven Cre recombinase expression induced a significant reduction in C/EBPβ protein levels in the liver (Figure 2*B*). We confirmed that C/EBPβ staining in hepatocyte nuclei was dramatically reduced without affecting the expression of the protein in nonparenchymal cells (Figure 2*B*). Moreover, hepatocytes isolated from these mice showed a dramatic reduction in C/EBPβ protein staining under control conditions or after treatment with 50 mM ethanol (Figure 2*C*), further confirming specificity of the KO. *Cebpb* KO did not affect weight changes in these mice (Figure 2*D*). Both groups of mice showed similar reduction of alanine aminotransferase and aspartate aminotransferase after 4 weeks of stopping alcohol (Figure 2*D*), similar final liver/body weight ratios (Figure 2*E*), liver glucose levels (Figure 2*F*), and liver steatosis resolution (Figure 2*G*).

**Figure 3:**
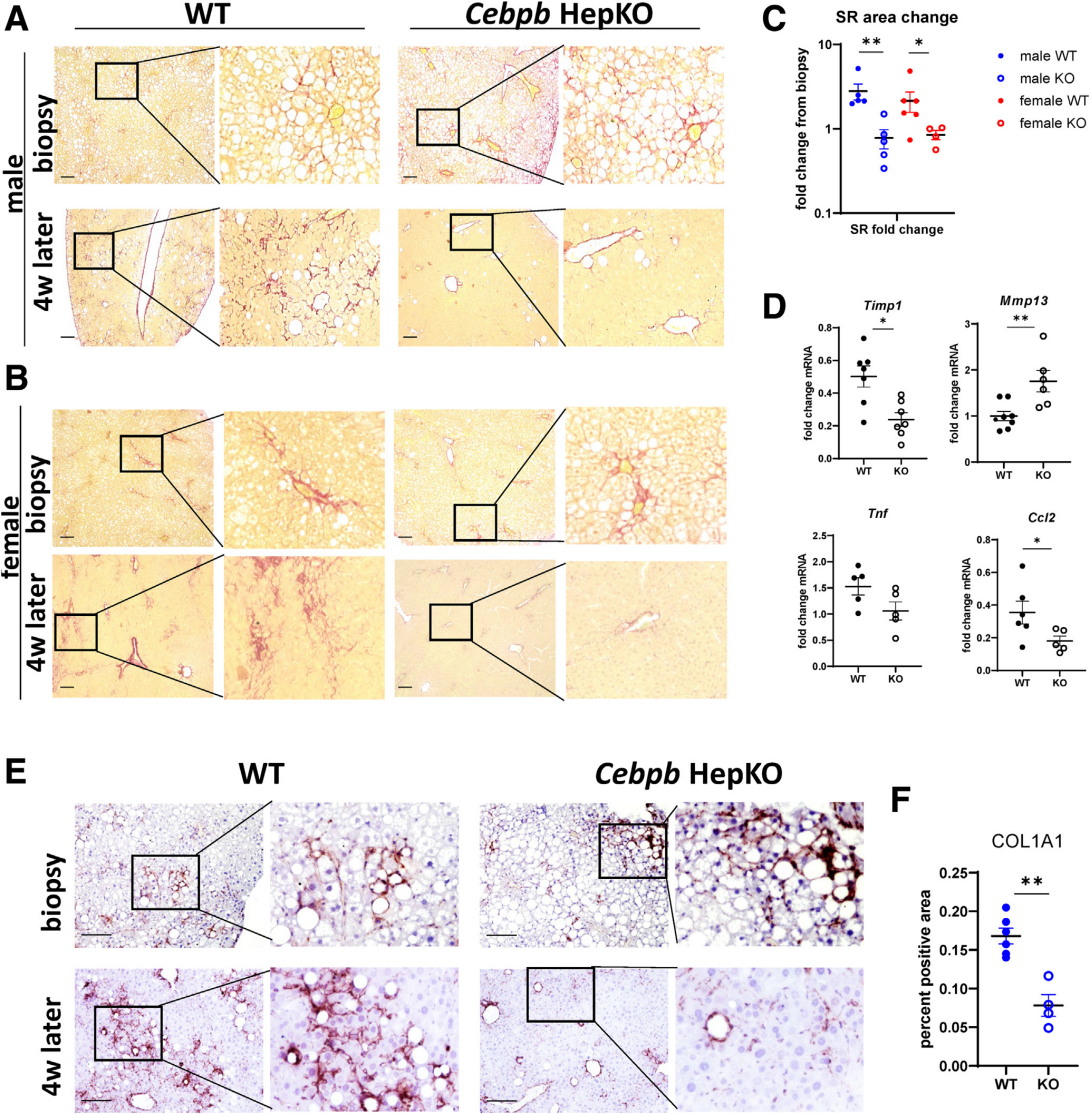
**Hepatocyte-specific *Cebpb* knockout promotes liver fibrosis resolution after alcohol cessation.** Seven- to 8-week-old *Cebpb* fl/fl mice were fed ad libitum WDA for 20 weeks then liver biopsy was collected, and mice were placed on chow diet with plain water for total of 4 weeks. One day later after biopsy, mice received 10^11^ gc/mouse of AAV-TBG-Cre or AAV-TBG-control. (*A, B*) Representative images of Sirius red staining from corresponding biopsy and livers after 4 weeks of resolution. (*C*) Fold change in Sirius red positive area from corresponding biopsy in each individual mouse. N ≥3 per group. ∗*P* < .05, ∗∗*P* < .01. (*D*) Relative mRNA expression (fold change from the biopsy) in WT and KO mice at 4 weeks of resolution. n ≥3 per group. ∗*P* < .05, ∗∗*P* < .01. (*E*) Representative images from corresponding biopsy and livers after 4 weeks of resolution of COL1A1 staining in WT or hepatocyte-specific KO mice. (*F*) COL1A1-positive area in WT and KO mice after 4 weeks. n = 3, ∗∗*P* < .01. Scale bar 100 μm.

We found that mice that received the AAV-control (wild-type [WT]) had no change in fibrosis levels between the biopsy and the explanted liver section after 4 weeks of abstinence (Figure 3*A* and *B*). This was similar to our previous observations.^13^ In contrast *Cebpb* KO promoted fibrosis resolution in males and females (Figure 3*A-C*). Differences in fibrosis correlated with gene expression changes associated with the fibrosis resolution phenotype, including increased MMPs, a decrease in the MMP inhibitor *Timp1*, and reduced inflammation-associated gene expression (Figure 3*D*). Better fibrosis resolution was associated with reduced COL1A1 staining in *Cebpb* KO mice after 4 weeks of alcohol cessation (Figure 3*E* and *F*).

Taken together C/EBPβ KO at the time of alcohol cessation promoted fibrosis resolution in WDA mice after WD and alcohol cessation without affecting other parameters, such as weight, injury, or liver steatosis.

### C/EBPβ-Mediated Fibrosis Resolution is Alcohol Specific

**Figure 4:**
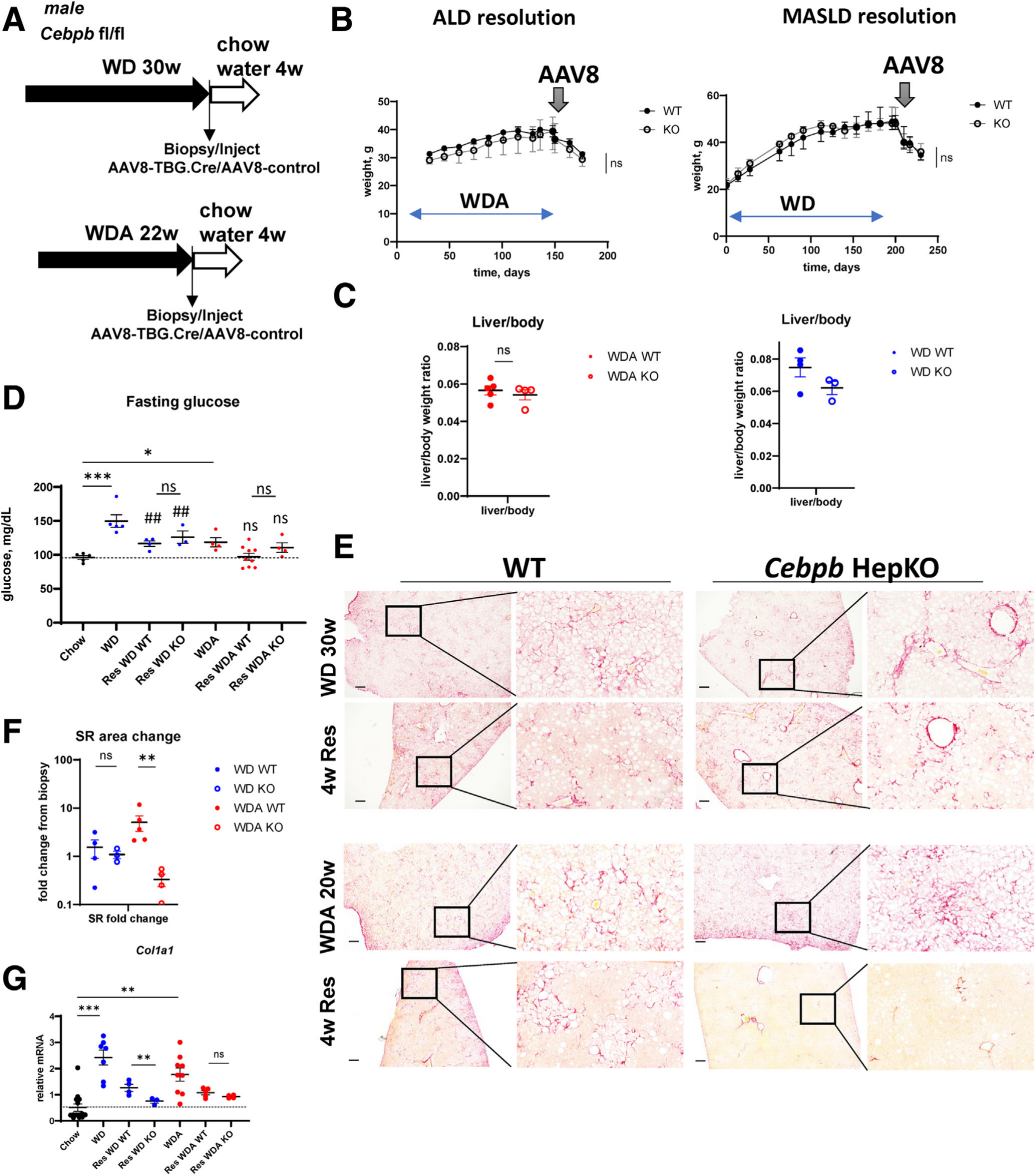
**The role of C/EBPβ in fibrosis resolution is alcohol specific.** (*A*) Seven- to 8-week-old *Cebpb* fl/fl mice were fed ad libitum WD for 30 weeks (metabolic dysfunction–associated steatotic liver disease [MASLD] model), or WDA for 22 weeks (ALD model) then liver biopsy was collected, and mice were placed on chow diet with plain water for total of 4 weeks. One day later after biopsy, mice received 10^11^ gc/mouse of AAV-TBG-Cre or AAV-TBG-control. (*B*) Weight change in these mice. (*C*) Liver to body weight ratios in mice at the end of the experiment. (*D*) Fasting glucose levels. (*E*) Representative images of Sirius red staining from corresponding biopsy and livers after 4 weeks of resolution. (*F*) Fold change in Sirius red positive area from corresponding biopsy in each individual mouse. n ≥3 per group. ∗∗*P* < .01. (*G*) Col1a1 mRNA levels in these mice. n ≥3 per group. ∗*P* < .05; ∗∗*P* < .01; ∗∗∗*P* < .001. Scale bar 100 μm. ns, not significant.

To assess whether the C/EBPβ role in fibrosis resolution is alcohol specific, we compared WDA (22 weeks of feeding, ALD model) with WD alone model (30 weeks of feeding, metabolic dysfunction–associated steatotic liver disease model) models of liver disease (Figure 4*A*). These result in similar liver fibrosis levels. For both groups of mice, we collected liver biopsies at the end of the feeding and injected AAV-TBG.Cre to induce *Cebpb* KO. Mice were then placed on chow diet with plain water for 4 weeks (Figure 4*A*). We found that KO did not affect weight changes in these mice (Figure 4*B*), liver to body weight ratio (Figure 4*C*), or fasting glucose levels (Figure 4*D*). In addition, C/EBPβ KO did not affect fibrosis resolution in WD fed mice, whereas it promoted fibrosis resolution in WDA group (Figure 4*E* and *F*), suggesting that C/EBPβ prevents fibrosis resolution specifically when alcohol was present.

Interestingly, we found that HSC activation markers, such as *Col1a1* mRNA, were reduced during ALD and metabolic dysfunction–associated steatotic liver disease resolution similarly in WD and WDA groups. However, in the WDA group we saw no effect of *Cebpb* KO, whereas in the WD group *Cebpb* KO showed greater reduction in *Col1a1* (Figure 4*G*). These data suggest that HSC activation changes are not the primary mechanism preventing fibrosis resolution in this model, and C/EBPβ KO promotes fibrosis resolution independently of HSC activation, perhaps by increasing matrix degradation. This agrees with our previous data demonstrating that C/EBPβ in hepatocytes had no direct effect on HSC activation.^32^

### C/EBPβ Loss Induced Profound Transcriptional Changes in the Liver After Alcohol Cessation

**Figure 5:**
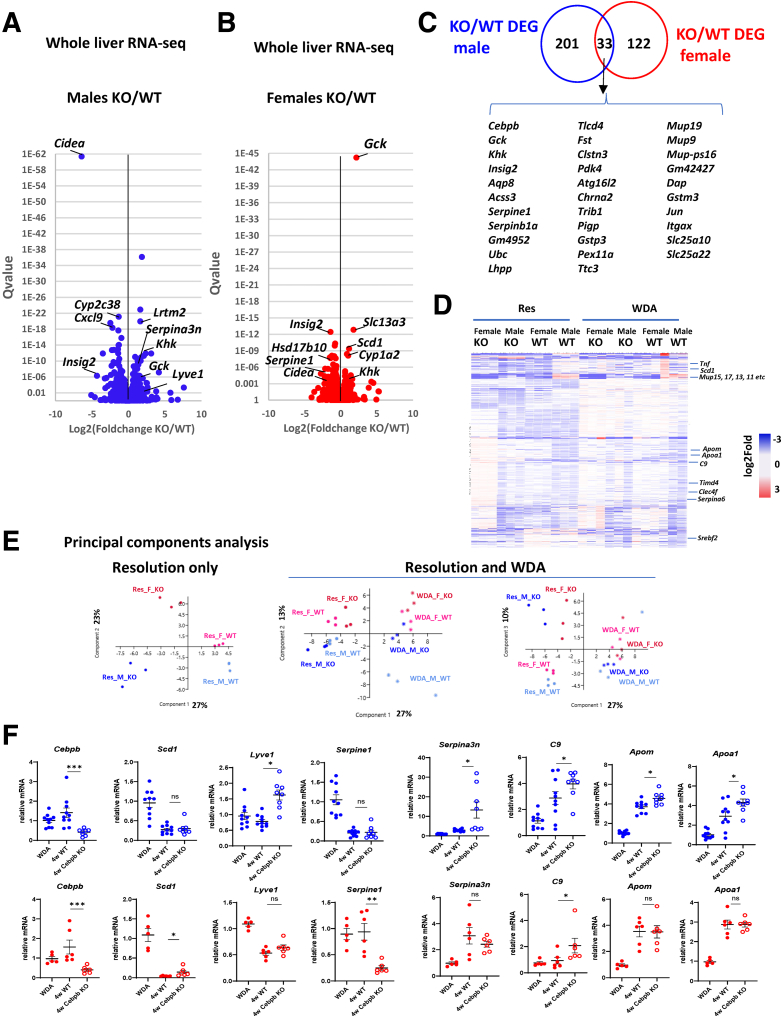
**Hepatocyte-specific *Cebpb* knockout controls metabolic gene transcription in the liver during ALD resolution.** (*A, B*) Volcano plot of differentially regulated genes in WT and KO male and female mice after 4 weeks of stopping alcohol. n = 3 mice per group. (*C*) Number of differentially expressed genes (Q-value < 0.01) in males and females. (*D*) Common and unique differentially expressed genes regulated by C/EBPβ during ALD development and resolution. (*E*) Principal components analysis of gene expression in WT and KO, male and female mice fed WDA and at 4 weeks after alcohol cessation. (*F*) Gene expression changes in WT and KO male (*top, blue*) and female (*bottom, red*) mice. ∗*P* < .05; ∗∗*P* < .01; ∗∗∗*P* < .001. ns, not signifcant.

To assess the mechanism of C/EBPβ-dependent ALD fibrosis resolution, we performed whole liver mRNA RNA-sequencing (RNA-seq) analysis in male (Figure 5*A*) and female (Figure 5*B*) WT and hepatocyte-specific *Cebpb* KO mice. Among 356 significantly (Q <0.01) differentially regulated genes we found only 33 were common between males and females including *Cebpb* itself and such genes as *Cidea*, *Khk*, *Gck*, and *Isgig2* highlighting the role of C/EBPβ in glucose and lipid metabolism (Figure 5*C*). When compared C/EBPβ-dependent genes during ALD resolution with C/EBPβ-dependent genes during ALD development (Figure 5*D*), we found that there was an overlap between genes regulated by C/EBPβ during ALD development and resolution. This further confirms the initial prediction of continuous activation of C/EBPβ induced by alcohol. In addition, we found many genes uniquely regulated by C/EBPβ during ALD resolution. Principal component analysis further highlighted the difference in C/EBPβ function during ALD development and resolution (Figure 5*E*). Combined WDA and resolution group analysis shows that about 27% of variance (PC1) is explained by the difference between WDA and resolution, whereas 13% (PC2) is caused by sex differences, and 10% (PC3) is caused by C/EBPβ KO during resolution, but not during ALD development. We confirmed gene expression changes of several differentially regulated genes by quantitative polymerase chain reaction analysis in larger number of liver samples (Figure 5*F*).

**Figure 6:**
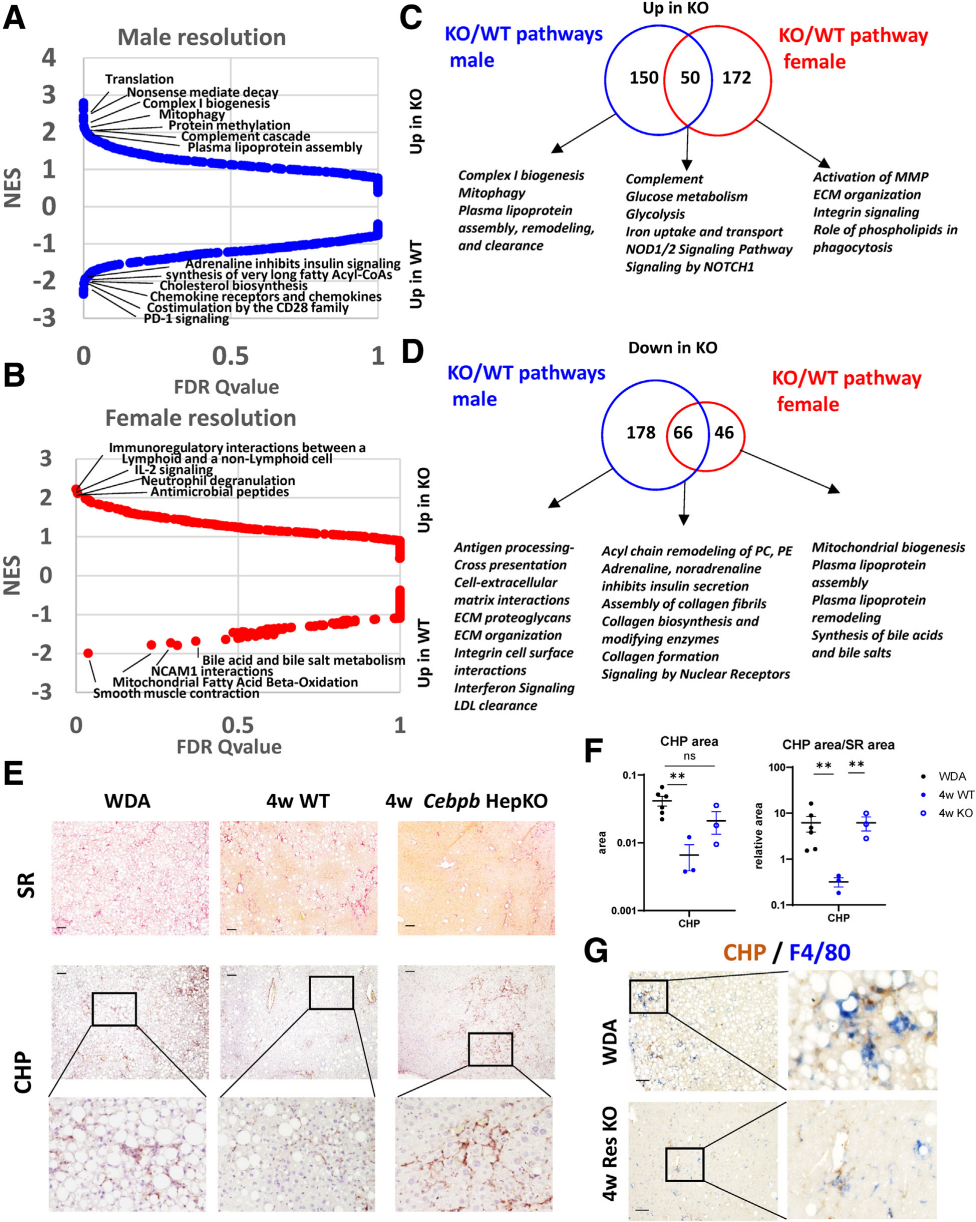
**Hepatocyte-specific *Cebpb* knockout controls collagen remodeling in the liver during ALD resolution.** (*A, B*) Pathway enrichment in differentially regulated genes using Gene Set Enrichment analysis regulated by C/EBPβ in males and females as indicated. (*C, D*) Common and unique pathways activated (*top*) and inhibited (*bottom*) in KO mice. (*E, F*) CHP staining in the livers of mice fed WDA or at 4 weeks after alcohol cessation. Biotin-conjugated CHP was incubated with liver sections overnight and visualized with streptavidin–horseradish peroxidase. (*G*) CHP colocalization with F4/80. Scale bar 100 μm. ∗∗*P* < .01. ns, not signifcant.

Top differentially regulated pathways in male and female mice (Figure 6*A* and *B*) highlight sex differences in C/EBPβ-dependent regulation of immune pathways and energy homeostasis. A few of the common pathways between males and females were associated with activation of glucose metabolism, iron uptake, complement cascade, and down-regulation of several pathways associated with collagen assembly and modification (Figure 6*C* and *D*) suggesting that some of these pathways could be involved in improved fibrosis resolution in these mice. Sex-specific pathways included activation of MMPs in females and reduced ECM related signaling in males that could further contribute to fibrosis changes in KO mice in a sex-specific way resulting in improved fibrosis resolution in both male and females.

To assess the collagen dynamics in WT and *Cebpb* KO mice, we used a collagen hybridizing peptide (CHP). The triple helix is the hallmark protein structure of collagen. During tissue remodeling, the triple helical collagen molecules are degraded by proteases (MMPs) and become unfolded. CHP can specifically bind to such denatured collagen strands through hydrogen bonding. We found that in WDA samples there is an active collagen remodeling, which significantly reduced at 4 weeks after alcohol cessation (Figure 6*E* and *F*). In contrast collagen remodeling was significantly elevated in C/EBPβ KO mouse livers compared with WT control animals and was similar to WDA levels, suggesting that active collagen remodeling contributes to fibrosis resolution in these mice. Moreover, because macrophages are known for their ability to promote fibrosis resolution, we examined colocalization between CHP staining and F4/80 (Figure 6*G*). We found that macrophages colocalized with the sites of collagen remodeling in WDA fed as well as *Cebpb* KO mice at 4 weeks after alcohol cessation, suggesting that macrophages contribute to fibrosis resolution in KO mice.

### C/EBPβ Modulates Hepatocyte Macrophage Crosstalk

**Figure 7:**
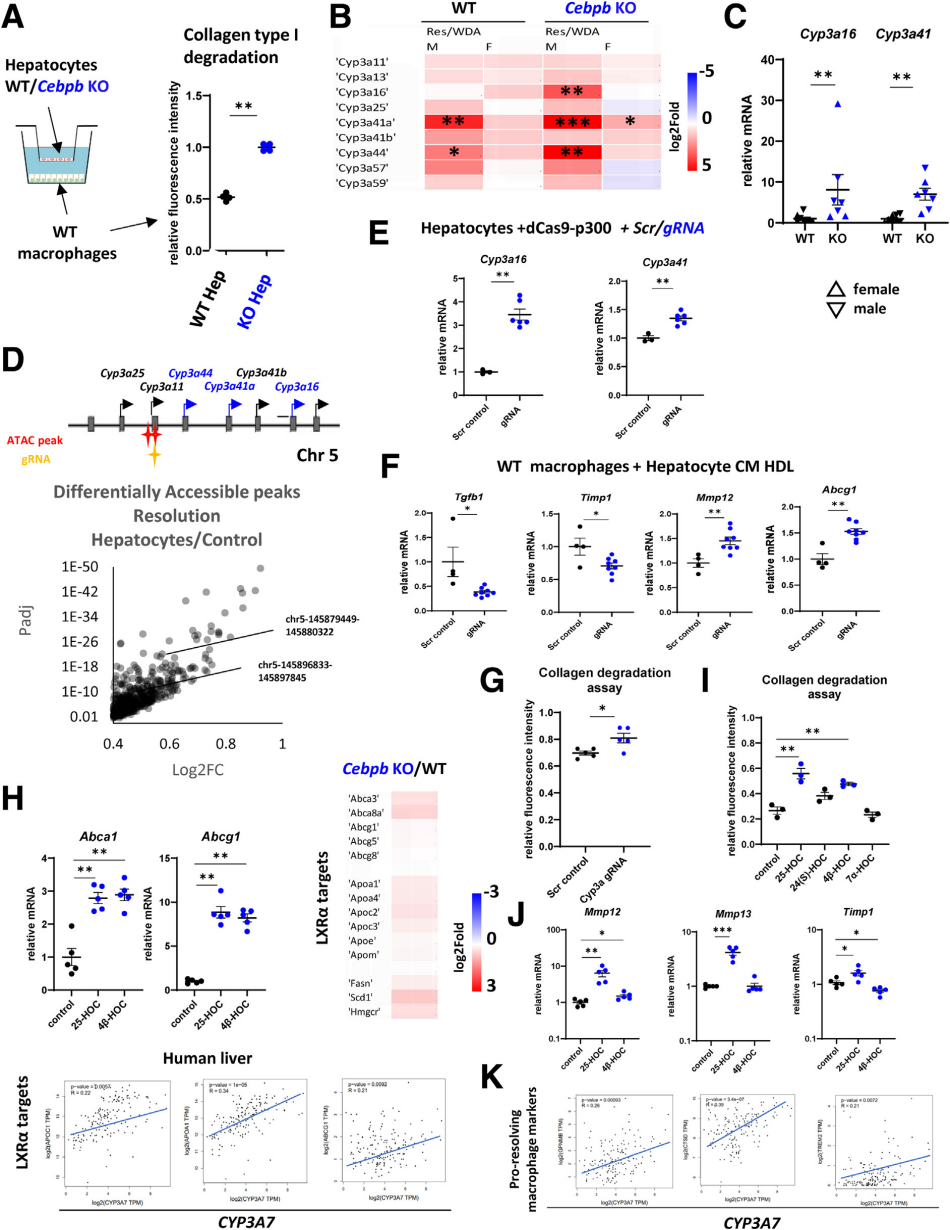
**C/EBPβ inhibition promotes proresolving phenotype in liver macrophages.** (*A*) WT or *Cebpb* KO hepatocytes were cocultured with WT liver macrophages in a Transwell coculture system. Macrophage collagen degradation activity, n = 4 per group. ∗∗*P* < .01. (*B*) RNA-seq data of Cyp3a gene expression fold change in WT mice during resolution (Res/WDA) and *Cebpb* KO mice during resolution (KO_Res/WDA). (*C*) Relative mRNA of *Cyp3a16* and *Cyp3a41* (*Cyp3a41a+ Cyp3a41b*) in WT and KO mice at 4 weeks after stopping alcohol. (*D*) Schematic of Cyp3a gene cluster showing ATAC peaks position and gRNA position. (*Bottom*) Differentially accessible peaks in hepatocytes from resolution condition compared with control hepatocytes. (*E*) Primary hepatocytes were transfected with dCas9-p300 vector and single gRNA targeting Cyp3a region or scrambled control. Relative mRNA expression in hepatocytes. (*F*) After 48 hours of incubation hepatocyte conditioned media was used for HDL isolation. Liver macrophages were treated with isolated HDL from hepatocytes expressing scr control RNA or Cyp3a targeting RNA. Relative mRNA in macrophages, n = 4–8 per group. ∗*P* < .05; ∗∗*P* < .01. (*G*) Macrophages were tested in a collagen degradation assay. n = 5 per group. ∗*P* < .05. (*H*) Macrophages were treated with 10 μM of corresponding oxysterols. (*Right*) Relative mRNA in macrophages, n = 4–8 per group. ∗∗*P* < .01. (*Right*) Whole liver LXRα target gene expression log2Fold (KO/WT). (*Bottom*) Correlation between CYP3A7 and LRXα target genes in human liver (TCGA + GTEx) samples. (*I*) Macrophages were treated with 10 μM of corresponding oxysterols. After 24 hours of culture macrophages were tested in a collagen degradation assay. (*J*) Relative mRNA in macrophages, n = 4–8 per group. ∗*P* < .05; ∗∗*P* < .01. (*K*) Correlation between CYP3A7 and proresolving macrophage marker genes in human liver (TCGA + GTEx) samples.

We next tested the effect of hepatocyte *Cebpb* KO on the phenotype of liver macrophages to examine cellular crosstalk involved in improved fibrosis resolution. We found that coculture with *Cebpb* KO hepatocytes promoted macrophage ability to degrade collagen, suggesting that hepatocyte to macrophage crosstalk is involved in fibrosis resolution mediated by C/EBPβ (Figure 7*A*). Previously we identified oxysterols as important mediators of hepatocyte-to-macrophage communication involved in fibrosis resolution in ALD.^13^ We examined gene expression of enzymes associated with oxysterol production in *Cebpb* KO mice and found that CYP3A family enzymes were elevated in KO mice compared with WT control animals. CYP3A enzymes metabolize a wide variety of substrates including cholesterol, bile acids, and triglycerides. They are important for oxysterol biogenesis of 4β-HOC and 25-HOC,^33^^,^^34^ the latter one we previously identified to promote ALD fibrosis resolution.^13^ RNA-seq data suggested that among CYP3A family of enzymes, several of them were elevated 2-fold or more in WT male and female mice after alcohol cessation (Figure 7*B*). In *Cebpb* KO mice expression of these genes were further elevated (Figure 7*B*). We measured mRNA levels of *Cyp3a16* and *Cyp3a41* in WT and KO mice at 4 weeks after alcohol cessation and found that both genes expression was elevated about 10-fold in KO mice compared with WT control subjects (Figure 7*C*). We identified 2 regions within CYP3A gene cluster of increased accessibility in mice during ALD resolution (Figure 7*D*), suggesting that this region might control increased gene expression of several of *Cyp3a* genes during ALD resolution. Next, we expressed a single gRNA targeting 1 of these regions together with a dCas9-p300 (epigenetic editor) construct in mouse hepatocytes. Compared with scrambled control, specific gRNA promoted expression of *Cyp3a16* and *Cyp3a41* genes in hepatocytes (Figure 7*E*). Because oxysterols are almost exclusively found in high-density lipoprotein (HDL)/low-density lipoprotein particles,^35^ we isolated HDL from conditioned media of these hepatocytes and used it to treat macrophages. HDL from CYP3A expressing hepatocytes induced a proresolving phenotype in macrophages to a greater extent than control HDL; it reduced *Tgfb1* and *Timp1* and increased *Mmp12* gene expression (Figure 7*F*). In addition, Cyp3a-HDL increased macrophage collagen degradation ability, suggesting that CYP3A elevation contributes to proresolving function of *Cebpb* KO hepatocytes (Figure 7*G*). Because oxysterols are known to activate LXRα activity, thus promoting macrophage proresolving phenotype, we assessed LXRα target gene expression in these macrophages. We found that enhanced collagen degradation correlated with elevated LXRα target gene *Abcg1* expression (Figure 7*F*). CYP3A enzymes can promote 4β-HOC and 25-HOC biosynthesis. To assess which of the 2 oxysterols is likely contributing to proresolving function of CYP3A we tested the function of synthetic compounds on macrophage phenotype and function. We found that 4β-HOC and 25-HOC induced LXRα target gene expression (Figure 7*H*). We next examined LXRα target gene expression in the livers of *Cebpb* KO mice compared with WT control animals. We found that LXRα target genes were uniformly activated in KO mice, suggesting that CYP3A activity promotes LXRα activation in vivo as well. In addition, we examined the relationship between CYP3A activity and LXRα activation in human liver. We found a strong positive correlation of human *CYP3A7* (homolog of *Cyp3a16*, *Cyp3a41*, and *Cyp3a44*) with several LXRα targets (Figure 7*H*). We found that 25-HOC was superior to 4β-HOC in promoting collagen degradation activity in macrophages. However, both oxysterols induced collagen degradation to greater extent compared with other tested oxysterols, 24(S)-HOC and 7α-HOC (Figure 7*I*). We further examined macrophage phenotype changes induced by oxysterol treatment. In vitro 25-HOC greatly induced *Mmp12* and *Mmp13* gene expression in macrophages, whereas 4β-HOC induced *Mmp12* only slightly (1.5-fold), and reduced *Timp1* gene expression, suggesting that CYP3A-mediated oxysterol production can directly stimulate proresolving macrophage markers (Figure 7*J*). Similarly in human samples we found positive correlations of *CYP3A7* with *GPNMB*, *TREM2*, and *CTSD*, markers of proresolving macrophages (Figure 7*K*),^36^, ^37^, ^38^ suggesting that CYP3A activity could contribute to fibrosis resolution in humans as well. Taken together CYP3A-mediated HDL remodeling likely contributes to hepatocyte–macrophage crosstalk involved in fibrosis resolution after alcohol cessation.

### Endothelial Dysfunction Drives C/EBPβ Up-regulation in ALD

**Figure 8:**
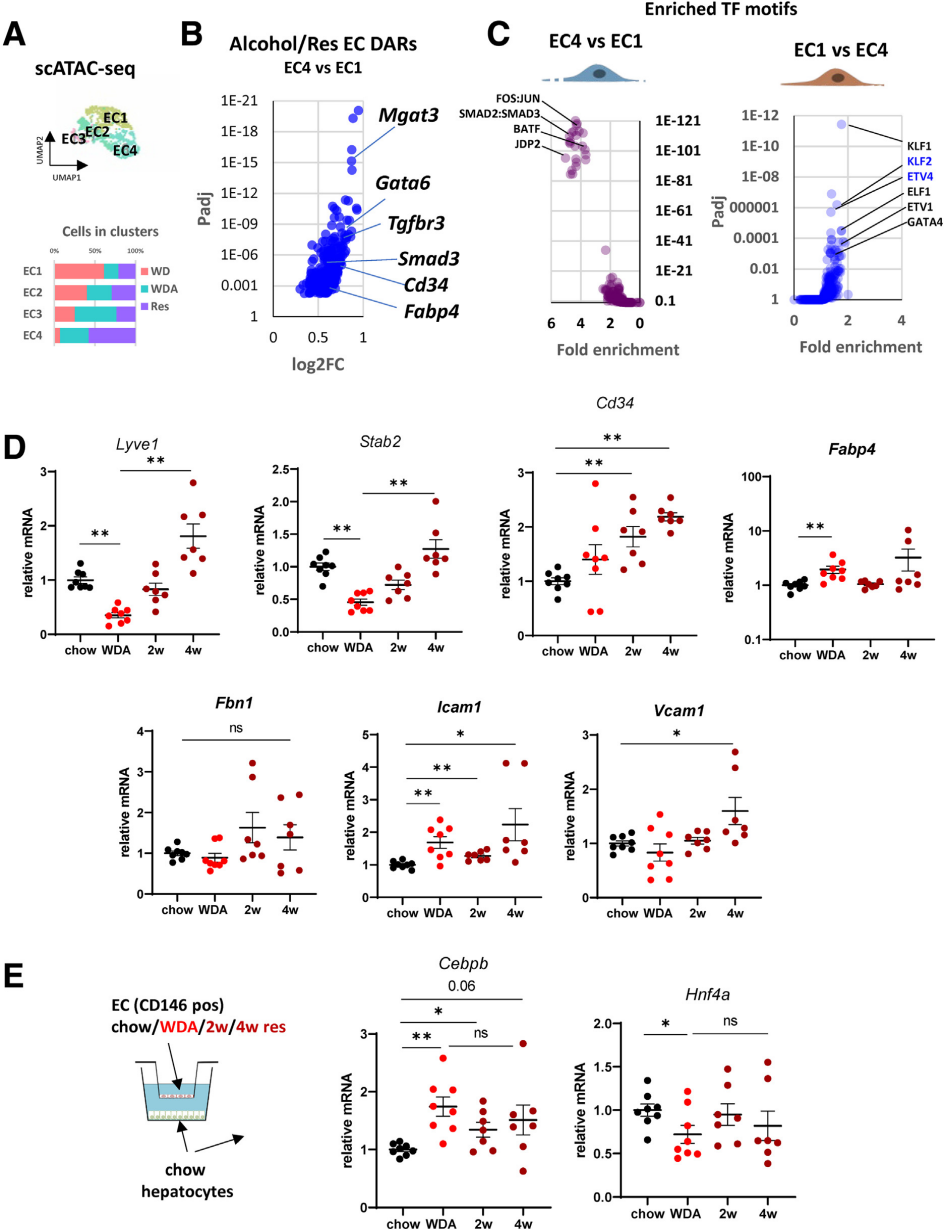
**Persistent endothelial dysfunction promotes C/EBPβ activation in ALD.** (*A*) Single cell ATAC-seq analysis of liver cells from mice fed WD for 16 weeks, WDA, and 16 weeks of WDA followed by 4 weeks of resolution (Res). UMAP clustering of endothelial cells and proportion of cells in clusters from each condition. (*B*) Top differentially accessible regions and associated genes. (*C*) Motif enrichment analysis. (*D, E*) Liver sinusoidal endothelial cells were isolated from mice fed chow diet, WDA, or WDA diet followed by 2–4 weeks of chow/water diet. (*D*) Relative mRNA in LSECs. n = 7–8 per group. ∗∗*P* < .01. (*E*) LSECs were cocultured with WT liver hepatocytes from chow fed mice in a Transwell coculture system. Relative mRNA in hepatocytes. n = 7–8 per group. ∗*P* < .05; ∗∗*P* < .01. ns, not signifcant.

Previously we identified endothelial dysfunction as 1 of the major drivers of C/EBPβ activation in the liver.^29^ Thus, we examined endothelial cell open chromatin landscape in control and alcohol-fed mice and in mice after alcohol cessation using our previously described scATAC-seq data set (Figure 8*A*).^13^ Under control conditions (WD with plain water), endothelial cells could be divided into 3 major clusters (EC1, EC2, EC3), which reflect liver zonation. Alcohol feeding resulted in formation of a new cluster (EC4). The EC4 cluster is characterized by a unique set of differentially accessible regions that are associated with such genes as *Tgfbr3*, *Gata6*, *Cd34*, and *Fabp4* (Figure 8*B*). EC4 closely resembles a dysfunctional defenestrated liver sinusoidal endothelial cells (LSEC) population described previously by the Iwakiri group and others.^39^, ^40^, ^41^, ^42^ We found that the EC4 population increased further and was predominant after 4 weeks of alcohol cessation suggesting that EC dysfunction persists after alcohol withdrawal and could contribute to poor disease resolution in ALD.

TF enrichment analysis identified AP-1 and SMAD2/3 as key drivers of EC4 epigenetic changes, whereas KLF1/2, ETV1/4, and GATA4 were involved in maintaining EC1 phenotype (Figure 8*C*). To test whether EC dysfunction persisted after alcohol cessation we isolated LSECs (CD146-positive liver cells) from mice on control chow diet, mice fed WDA for 20 weeks, and mice after 2 or 4 weeks of alcohol cessation (Figure 8*D*) and assessed gene expression. We found that alcohol reduced LSEC differentiation markers (*Lyve1*, *Stab2*) while increasing markers of LSEC dysfunction (*Fabp4*, *Icam1*, *Cd34*). After alcohol cessation *Lyve1* and *Stab2* expression returned to control levels, but in contrast, *Icam1*, *Vcam1*, and *Cd34* stayed elevated or further increased (Figure 8*D*) suggesting that endothelial dysfunction persists after alcohol cessation.

Next, we tested whether alcohol-induced LSEC dysfunction could promote *Cebpb* expression in hepatocytes. We found that coculture of isolated LSECs from alcohol-fed mice (either active drinkers or 2–4 weeks after resolution) induced *Cebpb* gene expression in hepatocytes from chow-fed animals (Figure 8*E*). WDA LSECs had the greatest effect on hepatocytes, both inducing *Cebpb* and reducing *Hnf4a* gene expression, whereas resolution LSECs had a partial effect on *Cebpb* induction, and no significant effect was observed for *Hnf4a*.

Taken together, we found that alcohol-induced LSEC dysfunction contributes to C/EBPβ activation in hepatocytes even after alcohol withdrawal.

### ANG-1 Supplementation Promotes Fibrosis Resolution

**Figure 9:**
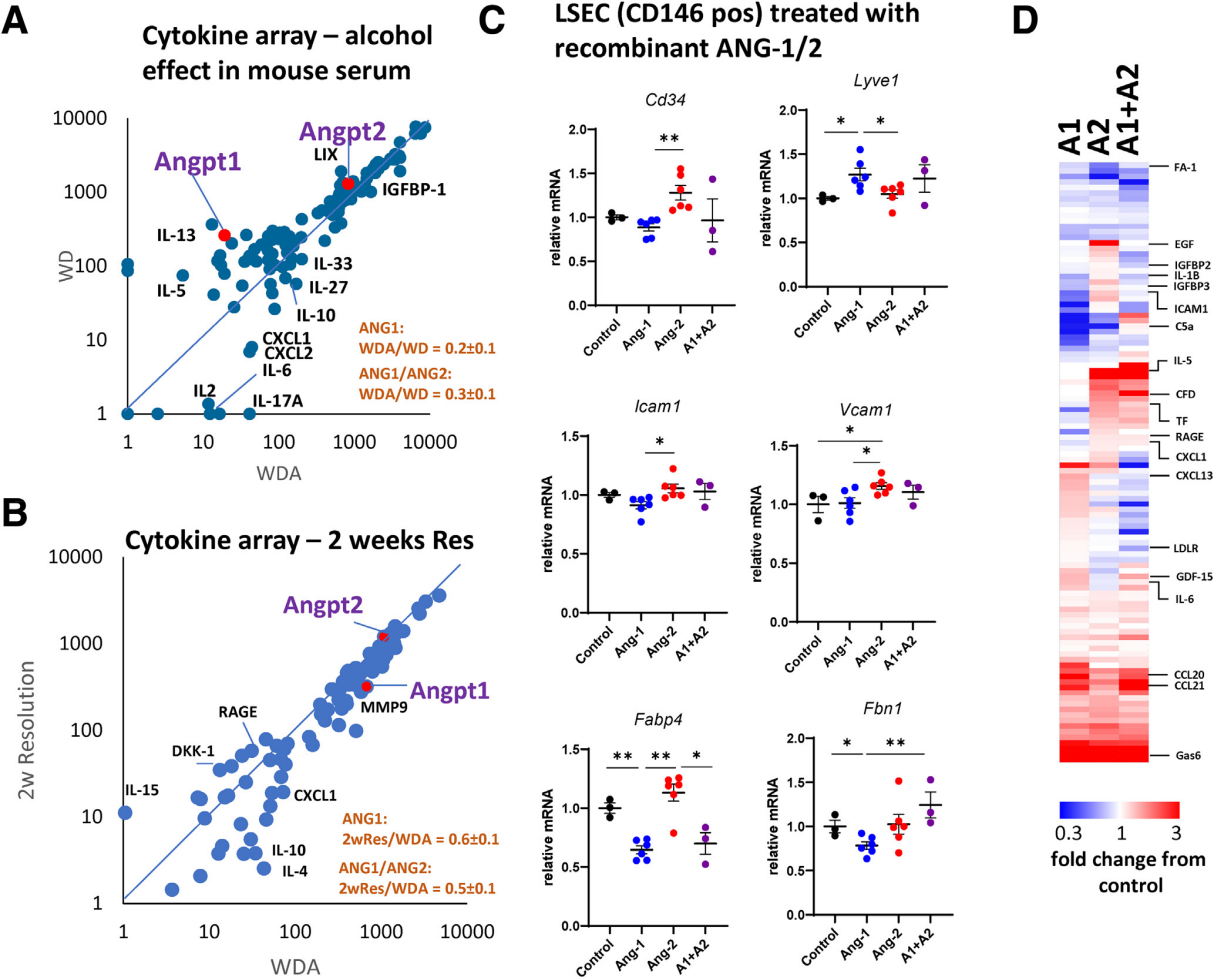
**Angiopoietin imbalance promotes endothelial dysfunction.** (*A*) Serum samples from WD and WDA fed mice after 12 weeks of feeding (pooled from n = 3 mice each) were used for cytokine array. Relative abundance of cytokines and circulating molecules. (*B*) Serum samples from WDA fed mice after 20 weeks of feeding and mice after 2 weeks of alcohol cessation (pooled from n = 3 mice each) were used for cytokine array. Relative abundance of cytokines and circulating molecules. (*C*) Primary mouse LSECs were treated with recombinant Angiopoietin-1, or Angipoietin-2, or both (10 ng/mL each) or saline control. n = 3–6 per group. ∗*P* < .05; ∗∗*P* < .01. (*D*) Endothelial cell secretome changes after treatment with recombinant Angiopoietin-1, or Angipoietin-2, or both (10 ng/mL each) compared with saline control.

Among alcohol dysregulated TF in EC4 we identified KLF2 and ETV4 (Figure 8*C*), known TFs downstream of ANG-1-TIE1/2 signaling.^43^, ^44^, ^45^ To assess the role of ANGs we examined circulating signaling molecule levels using a cytokine array (Figure 9*A*). We found that alcohol feeding down-regulated serum ANG-1 levels while circulating levels of ANG-2 were not changed thus reducing ANG-1/ANG-2 ratio about 3-fold (Figure 9*A*). We found that during ALD resolution, ANG-1 levels remain low and even slightly reduced 2 weeks after alcohol cessation (Figure 9*B*). This suggests that ANG-1/ANG-2 decrease might play a role in alcohol-induced persistent LSEC changes.

To validate this hypothesis, we tested the effect of in vitro ANG-1 and ANG-2 treatment on LSEC gene expression changes (Figure 9*C*). Previously we showed that ANG-1/ANG-2 ratio in vitro controlled hepatocyte *Cebpb* gene expression in hepatocyte-LSEC coculture.^29^ We tested whether ANG-1/ANG-2 ratio also altered markers of endothelial dysfunction found in ALD mice. We found that increased ANG-1/ANG-2 ratio resulted in an increase of *Lyve1* and a decrease in *Cd34*, *Fabp4*, *Fbn1*, and *Icam1*, a pattern opposite of the alcohol-induced changes (Figure 9*C* compared with 8*D*). Altered EC phenotype correlated with altered EC secretome (Figure 9*D*). ANG-2 induced whereas ANG-1 suppressed factors, such as EGF, IGFBP2 and IGFBP3, CXCL1, and IL-1β, known to control hepatocyte differentiation and promote C/EBPβ activation.^46^, ^47^, ^48^, ^49^, ^50^ Taken together these data suggest that ANG-1/ANG-2 imbalance drives endothelial disfunction and C/EBPβ up-regulation.

**Figure 10:**
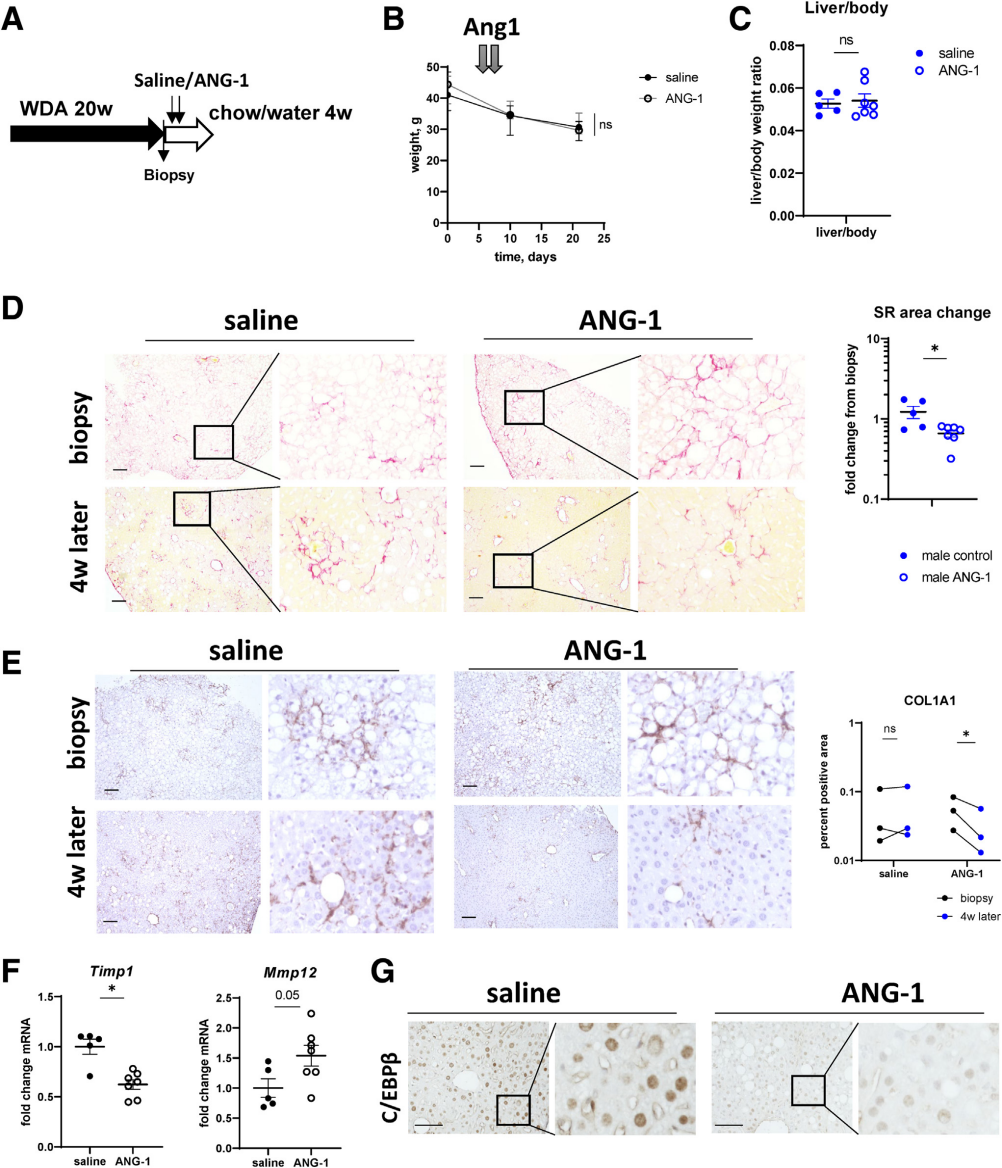
**ANG-1 supplementation promotes fibrosis resolution.** (*A*) Seven- to 8-week-old male mice were fed ad libitum WDA for 20 weeks then liver biopsy was collected, and mice were placed on chow diet with plain water for total of 4 weeks. One week after biopsy, mice received 3 μg/mouse of recombinant ANG-1 or saline control on 2 consecutive days. (*B*) weight change in these mice. (*C*) Liver to body weight ratio at the end of the experiment. (*D*) Representative images of Sirius red staining from corresponding biopsy and livers after 4 weeks of resolution. Fold change in Sirius red positive area from corresponding biopsy in each individual mouse. n ≥3 per group. ∗*P* < .05. (*E*) Representative images of COL1A1 staining from corresponding biopsy and livers after 4 weeks of resolution. Change in COL1A1-positive area from corresponding biopsy in each individual mouse. n = 3 per group. ∗*P* < .05. (*F*) Relative liver mRNA in mice at the end of the experiment. n = 5–7 per group. ∗*P* < .05. (*G*) Representative images of C/EBPβ staining in livers after 4 weeks of resolution. Scale bar 100 μm. ns, not signifcant.

Next, we tested whether ANG-1 supplementation could promote ALD resolution in vivo. We fed mice WDA for 20 weeks and subsequently placed them on chow diet with plain water for 4 weeks. After 1 week of alcohol withdrawal, we treated mice with 3 μg of recombinant ANG-1 on 2 consecutive days (Figure 10*A*). ANG-1 treatment did not affect weight and liver/body weight ratios of the mice (Figure 10*B* and *C*). In contrast, ANG-1 treatment promoted fibrosis resolution (Figure 10*D* and *E*). and promoted proresolving gene expression changes in the liver (MMP/tissue inhibitor of metalloproteinases) (Figure 10*F*). Improved fibrosis resolution correlated with reduced C/EBPβ expression in the liver (Figure 10*G*), suggesting that ANG-1-mediated C/EBPβ down-regulation contributed to improved fibrosis resolution in these mice.

## Discussion

Environmental exposures, such as alcohol and high-fat diet, can promote long-lasting epigenetic changes that can affect disease progression. Early epigenetic changes induced in response to environmental changes are likely involved in modulating the expression of genes important for adaptation to environmental exposures.^51^, ^52^, ^53^ However, growing evidence suggests that long-term accumulation of epigenetic changes because of chronic exposures results in harmful gene expression changes that can promote disease progression.^54^, ^55^, ^56^, ^57^, ^58^, ^59^, ^60^ In the case of ALD, alcohol-induced epigenetic changes promote liver inflammation,^54^^,^^57^^,^^58^^,^^61^ loss of hepatocyte differentiated function,^53^^,^^62^, ^63^, ^64^ liver fibrosis,^65^, ^66^, ^67^ and eventually hepatocellular carcinoma.^59^^,^^62^^,^^68^

Liver disease progression to advanced stages is associated with accumulation of epigenetic marks, such as histone modifications and DNA methylation, and additive effect of these marks makes the disease less reversible even after the exposure to harmful agents is over.^69^ In patients with ALD, recovery after alcohol cessation is not uniform and some patients progress to cirrhosis or develop decompensation while abstinent.^1^^,^^5^^,^^6^ There is, however, a limited understanding the molecular mechanisms of ALD resolution and further studies are necessary to define the drivers of resolution and the factors that prevent resolution of ALD with abstinence. Recently, we identified that accumulation of alcohol-induced epigenetic changes in hepatocytes prevented fibrosis resolution after alcohol cessation in a mouse model of ALD.^13^ We found that alcohol-induced KDM5B- and KDM5C-dependent epigenetic changes produce alterations in cell-cell communication in the liver that persist after alcohol withdrawal and prevent a proresolving phenotype shift in nonparenchymal cells, such as macrophages. Inhibition of KDM5 demethylases promoted fibrosis resolution in part through altered cholesterol metabolism and generation of oxysterol bile acid intermediates suggesting that active lipid metabolites are important for disease resolution. A recent clinical study that explored a metabolic signature of cirrhosis resolution in patients after the removal of underlying cause found circulating levels of taurocholic acid and taurochodeoxycholic acid as predictors of fibrosis regression.^70^ Taken together animal studies and clinical data suggest that metabolic functions of hepatocytes, specifically those related to cholesterol metabolic pathways, determine the capacity for fibrosis regression after alcohol cessation.

In this work we took an unbiased approach to define the TF-drivers of alcohol-induced changes in liver cells using our previously published scATAC-seq dataset. Specifically in hepatocytes, we identified that epigenetic changes were associated with activation of the C/EBPβ TF, and this persisted after alcohol cessation. The role of C/EBPβ in metabolic-associated liver disease or ALD has not been previously recognized. C/EBPβ has a known function in liver regeneration after partial hepatectomy and recently we identified C/EBPβ as a key driver in acute-on-chronic liver failure, where it antagonizes HNF4α-mediated transcription and drives the suppression of hepatocyte metabolic and synthetic function.^29^ In addition, C/EBPβ is known to regulate inflammatory responses, acute phase response gene expression, growth hormone response, and glucose signaling.^71^ Recently we demonstrated that C/EBPβ promotes liver fibrosis development in male mice fed WD with alcohol in the drinking water via alteration in intrahepatic cell-cell communication.^32^

In this study we found *Cebpb* KO at the time of alcohol cessation induced fibrosis resolution suggesting that persistent C/EBPβ activation is partly responsible for poor disease resolution in mice. We found that improved fibrosis resolution in KO mice was alcohol specific, because we did not observe C/EBPβ-mediated fibrosis resolution in a metabolic dysfunction–associated steatotic liver disease model, and it was associated with changes in cholesterol/bile acid metabolism and increased expression of the CYP3A family of enzymes, suggesting that these pathways might be involved in fibrosis resolution. CYP3A enzymes metabolize a wide variety of substrates including cholesterol, bile acids, and triglycerides. *Cyp3a* KO mice have altered HDL, cholesterol, and bile acid levels.^72^^,^^73^ They are important for oxysterol biogenesis of 4β-HOC and 25-HOC,^33^^,^^34^ which we showed before to promote ALD fibrosis resolution.^13^ We found that inducing CYP3A gene expression by targeting common enhancer resulted in altered HDL production, that can promote proresolving function in liver macrophages. This increased proresolving function is likely mediated by increased oxysterol production, as evidenced by elevated LXRα target gene expression in macrophages and in the livers of *Cebpb* KO mice. Circulating oxysterols are almost exclusively found in HDL.^35^ Thus, CYP3A induction in *Cebpb* KO hepatocytes likely promotes HDL lipid remodeling to stimulate HDL proresolving functions. However, a specific role of CYP3A-produced oxysterols or other metabolites will need further validation.

Other pathways that we found to be regulated by C/EBPβ during disease resolution included glucose metabolism related pathway. Among top differentially regulated genes in *Cebpb* KO mice was *Gck*. Previous studies identified that the rs1260326 variant of the *GCKR* gene encoding for glucokinase regulator was associated with a lower probability of fibrosis regression in patients with liver cirrhosis.^70^ These data suggest that these metabolic genes could be not only correlating with fibrosis resolution but also functionally involved in resolution process.

Previous studies have shown that C/EBPβ activation is usually transient; thus, we assessed the possible mechanisms that could be responsible for persistent C/EBPβ activation in ALD. Using alcohol treatment of isolated primary hepatocyte, we found that alcohol can only moderately (1.3-fold) increase C/EBPβ protein levels, suggesting that cell intrinsic mechanisms only partially contribute to C/EBPβ elevation. Recently we have demonstrated that in an acute-on-chronic liver failure model, C/EBPβ activation was mediated by endothelial ANG signaling.^29^ ANG-1 and -2 are key regulators of endothelial cell fate and function.^74^, ^75^, ^76^ Increased ANG-2/ANG-1 ratio is a well-known feature of liver disease.^77^^,^^78^ Both ANG-1 and ANG-2 competitively bind TIE1 and TIE2 receptors on endothelial cells and activate ligand-specific downstream signaling.^79^^,^^80^ ANG-2, in addition, has TIE-independent functions.^81^, ^82^, ^83^, ^84^ Previously we found that ANG-1 and ANG-2 alter endothelial cell-hepatocyte crosstalk and regulate *Cebpb* expression.^29^

In this work we found that alcohol-induced epigenetic changes in LSECs were mediated by ANG-1 and ANG-2 imbalance, which correlated with C/EBPβ activation. Moreover, the endothelial cell epigenetic state did not revert to the control state after alcohol cessation, and this correlated with persistent C/EBPβ activation in mice up to 4 weeks after alcohol withdrawal. LSEC are key players in liver homeostasis and endothelial dysfunction alone can drive liver fibrosis development.^39^^,^^85^ We found that LSECs isolated from mice after 2 and 4 weeks of stopping alcohol showed markers of endothelial dysfunction and were able to induce *Cebpb* expression, suggesting that persistent epigenetic changes in LSECs contribute to C/EBPβ activation. We were able to reduce endothelial dysfunction by supplementing mice with recombinant ANG-1, which reduced C/EBPβ expression and promoted fibrosis resolution. Taken together we showed that ANG-1/ANG-2 imbalance induced by alcohol drives long-lasting endothelial cell changes that alter EC-hepatocyte signaling and promote C/EBPβ activation, which in turn promotes fibrosis development and prevents fibrosis resolution even after alcohol withdrawal.

Interestingly we found that C/EBPβ protein expression was further induced during liver disease resolution compared with WDA group, which could be caused by high-fat diet and alcohol withdrawal. However, the mechanism of this induction is not clear. It must be independent of LSEC-mediated signaling, because we did not find this increase in EC-hepatocyte coculture. We speculate that this increase could be caused by glucose signaling in hepatocytes, because C/EBPβ is known for its role in glucose sensing in liver regeneration models.^15^

Our data suggest that C/EBPβ is a promising treatment target for ALD. Because C/EBPβ levels are low under normal conditions C/EBPβ inhibition could be a promising therapeutic strategy to promote fibrosis resolution in patients with ALD after alcohol cessation. Several peptides have been shown to inhibit C/EBPβ TF activity, specifically Dpep, Bpep, and ST101.^71^^,^^86^^,^^87^ ST101 is currently in a clinical trial for glioma. Future studies are necessary to define the potential of targeting C/EBPβ in ALD resolution.

In summary, this work illustrates how persistent alcohol-induced changes in endothelial cell function and hepatocyte epigenetic state can trigger a series of alterations in cell-cell crosstalk phenomena. These lead to persistent C/EBPβ expression in hepatocytes and consequent hepatocyte-driven loss of the antifibrotic restorative phenotype in macrophages. Although many details of these interactions remain to be determined, they open several potential possible therapeutic interventions to improve the resolution of ALD after alcohol cessation.

## Materials and Methods

### Mice

*Cebpb* floxed mice (BALB/cJ-Cebpb^tm1.1Elgaz^) were obtained from Jackson Laboratory and backcrossed for 7 generations to the C57BL6/J background. All mice were housed in a temperature-controlled, specific pathogen-free environment with 12-hour light-dark cycles. All animal handling procedures were approved by the Institutional Animal Care and Use Committee at the University of Kansas Medical Center (Kansas City, KS).

For fibrosis induction mice were treated with 200 mg/L of TAA in the drinking water for 2 months. Recombinant ANG-1 or saline control was injected intraperitoneally at 3 μg/mouse.

### WDA Model

For the previously described WDA model,^30^ both male and female mice were fed ad libitum Western diet (Research Diets, Inc, Cat# D12079B) and alcohol was given ad libitum in water. Mice received progressively increasing amounts of alcohol in water (3%, 10%, 15%, and 20% for 3 days each). After reaching 20%, mice continued for 12–18 weeks as indicated. Alcohol-containing water was changed twice weekly.

### Vectors

AAV-TBG-control and AAV-TBG-iCre were from VectorBiolabs (Malvern, PA) and were used at 1 x 10^11^ genome copies per mouse (Cre/control). dCas9-p300 plasmid was from Addgene (pcDNA-dCas9-p300 Core, Cat# 61357). Predesigned single-guide RNA was from IDT (Design ID: Mm.Cas9.CYP3A11.1.AA, chr 5 position 145879712).

### Biopsy

Small liver biopsies were collected as previously described.^88^ Mice were anesthetized with isoflurane; a small incision was made on the upper right side of the abdomen and the liver exposed. One lobe of the liver was carefully lifted, and a small piece of liver (3–5 mm) was excised with scissors. The tissue was immediately placed in zinc-formalin and RNA-later for further processing. The gap in the liver was closed with an absorbable hemostatic gelatin sponge (Vetspon #96002). The incision was closed with 5–0 absorbable surgical suture (Redilene Redisorb Fast Pro #VF493-M) and 7-mm wound-clips (Reflex 7 #203-1000). Mice were then injected with 1 mL saline (subcutaneously) and 1.0 mg/kg SR buprenorphine (subcutaneously). Mice were placed on a heating pad and monitored until fully awake from anesthesia; thereafter mice were monitored daily for the next 7 days. The wound-clips were removed on Day 8 after surgery.

### HDL Isolation

HDL was isolated using HDL Purification Kit (Cell Biolabs Inc) according to manufacturer’s instructions. HDL was dialyzed against phosphate-buffered saline (PBS) overnight and used at 20 μg/mL. HDL purity was confirmed by gel electrophoresis in denaturing and nondenaturing conditions.

### Antibodies

Anti-COL1A1 antibodies were from Cell Signaling (COL1A1 [E8I9Z] Rabbit mAb #91144). Anti-C/EBPβ antibodies were from SantaCruz (C/EBP beta Antibody [H-7]: sc-7962).

### Analysis of Blood Samples

Whole blood collected from the retroorbital vein of mice was used to measure prothrombin time (CoaguChek XS system, 04625315160, Roche). Serum was used to measure alanine aminotransferase (Pointe Scientific ALT Liquid Reagents, A7526150, Pointe Scientific) and aspartate aminotransferase (Pointe Scientific ALT Liquid Reagents, A7561450, Pointe Scientific).

### RNA-Seq

For RNA-Seq analysis, total RNA was isolated from the liver using the Qiagen RNA isolation kit. Three individual mice were used per condition. Library generation and sequencing was performed by BGI genomics services (BGI, Cambridge, MA). Twenty-seven samples were sequenced using the BGISEQ platform, generating an average of approximately 4.57G Gb bases per sample. HISAT was used to align the clean reads to the reference genome. Bowtie2 was used to align the clean reads to the reference genes. The average mapping ratio with a reference genome (GRCm38.p6) was 96.14% and 16,869 genes were identified. Differential gene expression was identified with DESeq2. RNA-seq raw data can be found under GSE276692.

### Cell Isolation

Liver cells were isolated by a modification of the method described by Troutman et al.^89^ Mouse livers were digested by retrograde perfusion with Liberase via the inferior vena cava. The dissociated cell mixture was placed into a 50-mL conical tube and centrifuged twice at 50 *g* for 2 minutes to pellet hepatocytes. The nonparenchymal cells–containing cell supernatant was further used to isolate KC, LSEC, and HSC. The cell suspension was pelleted by centrifugation (700 *g*, 10 minutes, 4°C) and resuspended in PBS and OptiPrep (Sigma) to a final concentration of 17%. Afterward, 5 mL of the indicated suspension was placed in a 15-mL polystyrene conical centrifuge tube (BD Biosciences) and overlaid with 5 mL of a 9% OptiPrep solution followed by 2 mL PBS. After centrifugation at 1400 *g* for 20 minutes at 4°C with decreased acceleration and without breaks, the various cell-types were arranged according to their density. HSC were enriched in the upper cell layer, whereas KC and LSEC were separated as a second layer of higher density. Cell fractions were collected separately by pipetting. HSC purity greater than 99% was confirmed by retinoid-based fluorescence-activated cell sorting. The KC/LSEC fraction was pelleted, KCs and ECs were isolated with F4/80^+^ and CD146^+^ MicroBeads (MiltenyiBiotec), respectively, according to the manufacturer’s instructions. Cells were applied onto LS magnetic-activated cell sorting columns (MiltenyiBiotec), which were placed within the magnetic field of a magnetic-activated cell sorting separator and washed 3 times with magnetic-activated cell sorting buffer (MiltenyiBiotec). Cells were eluted and were then seeded into culture dishes. ECs were seeded on dishes coated with collagen-I.

### Transwell Coculture

For coculture experiments, primary LSECs were placed in cell inserts of 24-well Transwell (Corning Incorporated, Acton, MA; 0.4 μm pore size) at a seeding density of 5 × 10^4^/well. Cells were treated as indicated. Freshly isolated hepatocytes were seeded in bottom well at a seeding density of 1 × 10^5^/well. The cells were then cultured for 24 hours, and hepatocytes were harvested for RNA isolation. Alternatively, primary hepatocytes from *Cebpb* floxed mice were placed in cell inserts. Cells were transfected with Cre recombinase expressing vector or empty vector control. Freshly isolated liver macrophages were seeded in bottom well. The cells were then cultured for 24 hours, and macrophages were harvested for RNA isolation. Alternatively, macrophages were used for a collagen degradation assay, using DQ Collagen (ThermoFisher, Cat# D12060), type I From Bovine Skin, Fluorescein Conjugate, according to manufacturer’s instructions. Cells were incubated in 50 mM Tris-HCl (pH 7.6), 150 mM NaCl, 5 mM CaCl2, 0.5% agar, and 10 mg/L DQ Collagen for 2 hours at 37°C. Digestion product fluorescence was measured at 525 nm.

### Immunohistochemistry

Liver tissue sections (5 μm thick) were prepared from formalin-fixed, paraffin-embedded samples. Immunostaining on formalin-fixed sections was performed by deparaffinization and rehydration, followed by antigen retrieval achieved by heating in a pressure cooker (121°C) for 5 minutes in 10 mM sodium citrate, pH 6.0, as previously described.^62^ Peroxidase activity was blocked by incubation in 3% hydrogen peroxide for 10 minutes. Sections were rinsed 3 times in PBS/PBS-T (0.1% Tween-20) and incubated in Dako Protein Block (Dako, Carpinteria, CA) at room temperature for 1 hour. After removal of blocking solution, slides were placed into a humidified chamber and incubated overnight with a primary antibody diluted 1:300 in Dako Protein Block at 4°C. The antigen was detected using the SignalStain Boost IHC detection reagent (catalogue # 8114; Cell Signaling Technology, Beverly, MA), developed with diaminobenzidene (Dako), counterstained with hematoxylin (Sigma-Aldrich), and mounted.

### Real-Time Polymerase Chain Reaction

RNA was extracted from livers using the RNeasy Mini Kit (Qiagen). cDNA was generated using the RNA reverse transcription kit (Applied Biosystems, Cat.No 4368814). Quantitative real-time polymerase chain reaction was performed in a CFX96 real-time system (Bio-Rad) using specific sense and antisense primers (Table 1) combined with iQ SYBR Green Supermix (Bio-Rad) for 40 amplification cycles: 5 seconds at 95°C, 10 seconds at 57°C, and 30 seconds at 72°C. mRNA concentrations were calculated relative to *Actb*.

**Table 1:** Primers

mActb fwd	ATGTCACGCACGATTTCCCT	mHnf4a fwd	AGCAATGGACAGATGTGTGAGT
mActb rvs	CGGGACCTGACAGACTACCT	mHnf4a rvs	TTCAGATCCCGAGCCACTTG
mTnf fwd	CTGAGACATAGGCACCGCC	mCebpb fwd	TCACTTAAAGATGTTCCTGCGG
mTnf rvs	CAGAAAGCATGATCCGCGAC	mCebpb rvs	TGCTCGAAACGGAAAAGGTTC
mCol1a1 fwd	TGGCCAAGAAGACATCCCTG	mStab2 fwd	CCAGCTGGGTAAATGCAACA
mCol1a1 rvs	GGGTTTCCACGTCTCACCAT	mStab2 rvs	ATATGACGGCTGGTGTCCTC
mMmp9 fwd	CCCTGGAACTCACACGACAT	mLyve1 fwd	GTAGCAAACAGCCAGCACAG
mMmp9 rvs	TCACACGCCAGAAGAATTTGC	mLyve1 rvs	TTTGTTGCAAGTGGAGCAGC
mTimp1 fwd	GTAAGGCCTGTAGCTGTGCC	mIcam1 fwd	TCACCGTGTATTCGTTTCCG
mTimp1 rvs	AGCCCTTATGACCAGGTCCG	mIcam1 rvs	GGTGAGGTCCTTGCCTACTTG
mTgfb1 fwd	TACGTCAGACATTCGGGAAGC	mVcam1 fwd	GGGGGCCACTGAATTGAATCT
mTgfb1 rvs	TTTAATCTCTGCAAGCGCAGC	mVcam1 rvs	GGAAGCTGGAACGAAGTATCC
mCcl2 fwd	ACCTGGATCGGAACCAAATGAG	mCd34 fd	CTCTCTGCCTGATGAGTCTGC
mCcl2 rvs	GCTGAAGACCTTAGGGCAGAT	mCd34 rvs	CCTTGTGTAGAAGTCTCCGTGG
mMmp12 fwd	GTGGTACACTAGCCCATGCTT	mFbn1 fwd	CCCGGGAATTACCAGAGGGA
mMmp12 rvs	TCCACGTTTCTGCCTCATCAA	mFbn1 rvs	TGTTGCTCCCGAGATGTGTC
mMmp13 fwd	ATGAAGACCCCAACCCTAAGC	mFabp4 fwd	ATCGAATTCCACGCCCAGTT
mMmp13 rvs	ATGGCATCAAGGGATAGGGC	mFabp4 rvs	GGATTTGGTCACCATCCGGT

### Cytokine Array

Proteome Profiler Mouse Cytokine Array Kit (R&D Systems) detecting 111 mouse cytokines was used according to manufacturer’s instructions to test relative cytokine abundance in serum of mice fed WD or WDA for 12 weeks.

### Collagen Hybridizing Peptide

CHP conjugates were from Advanced BioMatrix. CHP was labeled with biotin (B-CHP, Catalog No. 5265-60UG) for avidin/streptavidin-mediated detection and was used at 20 μM concentration in 1% bovine serum albumin according to manufacturer’s instructions.

Briefly, sections were deparaffinized, blocked with Fish Serum Blocking Buffer (Thermo Fisher), followed by endogenous biotin blocking (biotin blocking kit, Thermo Fisher). CHP was thermally dissociated at 80°C, quenched to room temperature, and added to tissue section over night at 4°C. Tissue sections were then incubated with streptavidin–horseradish peroxidase (5 μg/mL) for 30 minutes and developed with diaminobenzidene (Dako), counterstained with hematoxylin (Sigma-Aldrich), and mounted.

### Statistics

Data were plotted and analyzed in Prism GraphPad. Comparison between different data sets was made using unpaired 2-tailed *t* test with Welch correction and 1-way analysis of variance with Tukey post hoc test. *P* < .05 was considered to be statistically different.
